# Spontaneous left pneumothorax revealing an atypical carcinoid tumor: a case report

**DOI:** 10.11604/pamj.2024.49.93.45209

**Published:** 2024-11-26

**Authors:** Hajar Arfaoui, Naima Ait Mouddene, Chaimaa Belhaj, Hajar Bamha, Salma Msika, Nabil Bougteb, Hassnaa Jabri, Wiaam Elkhattabi, Hicham Afif

**Affiliations:** 1 Department of Pulmonology and Respiratory Disease, University Hospital Center of Casablanca, Casablanca, Morocco

**Keywords:** Bronchial tumor, neuroendocrine tumor, atypical carcinoid tumor, pneumothorax, case report

## Abstract

The association between spontaneous pneumothorax and a bronchial tumor is rare, and even more so with an atypical carcinoid tumor. This rare and unexpected case highlights the importance of raising clinician awareness about investigating the underlying cause of pneumothorax through endoscopic exploration. A 52-year-old female was exposed to passive smoking by her husband for 20 years. She was treated for hypothyroidism for the past two years, pulmonary tuberculosis a year ago, and cardiac disorders for the past eight months. The patient presented to the emergency room with a spontaneous left pneumothorax secondary to an atypical bronchial carcinoid tumor. The diagnosis was established through thoracic CT and bronchoscopy. Treatment involved left pneumonectomy with follow-up.

## Introduction

Spontaneous pneumothorax is a common pleural effusion in pulmonology consultations, often due to the rupture of subpleural emphysematous blebs or bullae [1]. However, the association of spontaneous pneumothorax with a bronchial tumor is rare and even more so with a carcinoid tumor [1,2]. Generally, these tumors are asymptomatic in 75% of cases, but they can be accompanied by non-specific respiratory symptoms such as recurrent bronchopneumonia, persistent cough, hemoptysis, recurrent fever, chest pain, and primarily dyspnea [2]. Carcinoid syndrome is mostly encountered in advanced cases and can be complete or incomplete, presenting with symptoms like flushing, diarrhea, dyspnea, and wheezing. Each case of atypical carcinoid tumor is important for improving the understanding of the clinical features, treatment options, and prognosis of this disease. The publication of case reports on such rare diagnoses contributes to the medical knowledge base and can help in developing new treatment strategies and early detection methods.

## Patient and observation

**Patient information:** the patient was a 52-year-old female with no toxic habits, exposed to passive smoking by her husband for 20 years. She has been treated for hypothyroidism with levothyroxine (25 mg/day) for two years, for pulmonary tuberculosis a year ago and declared cured, and for rhythm disorders and right bundle branch block with beta-blockers for eight months. Additionally, the patient had recent exposure to tuberculosis in her household.

**Timeline:** her symptoms began four months ago, with the gradual onset of a dry cough, mild left subaxillary chest pain described as a prickling sensation, alternating diarrhea and constipation, and hot flashes. This developed in an afebrile context with a preserved general condition. Fifteen days later, the patient experienced worsening of the dry cough and an increase in the intensity of the pain, which became stabbing.

### Clinical findings

**Examination upon admission to the hospital:** the patient was in good general condition, overweight (BMI= 28 kg/m^2^), tachypneic at 26 cycles/min, afebrile, normotensive, normocardic, with normal oxygen saturation in room air (SpO_2_: 99%), and without signs of respiratory distress or cyanosis. Pulmonary examination revealed a syndrome of air effusion on the posterior side of the entire left hemithorax. Cardiovascular examination showed no signs of chronic cor pulmonale. Examination of other systems was normal.

**Figure 1:**
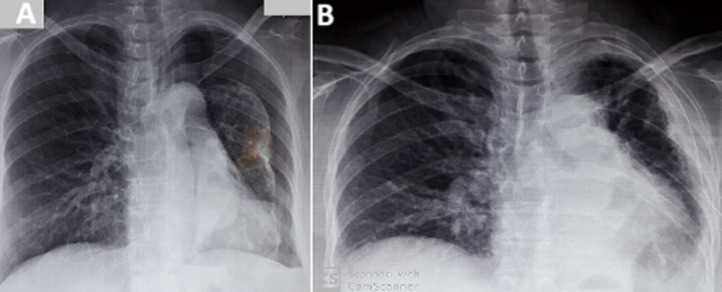
A) to the left, large left pneumothorax associated with blunting of the ipsilateral costophrenic angle and a slightly distended right lung; B) to the right re-expansion of the lung to the chest wall after thoracic drainage with the presence of an obstruction image at the level of the left main bronchus

**Figure 2:**
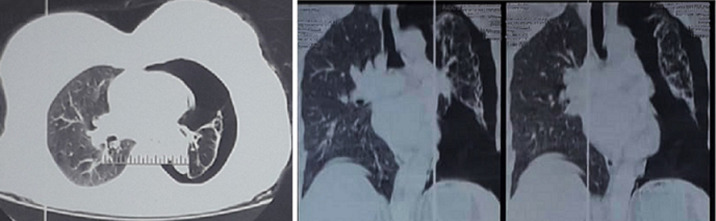
large left pneumothorax in axial and coronal views with a collapsed lung around the pulmonary hilum

**Radiological and biological assessment:** an emergency chest X-ray revealed a large left pneumothorax with a left pleural adhesion on a collapsed lung around the left pulmonary hilum (Figure 1). A chest computed tomography (CT) scan showed a left pneumothorax with a hypodense obstruction completely blocking the left main bronchus in its middle third, along with bronchial dilatations and septal thickenings (Figure 2). An emergency left axillary thoracic drainage was performed, with the lung returning to the chest wall (Figure 1). Post-drainage radiography showed an obstruction image in the left main bronchus, a retracted left lung, pleural thickening, and proximal areolar images. Complete blood count, liver, renal, and thyroid function tests were normal.

**Figure 3:**
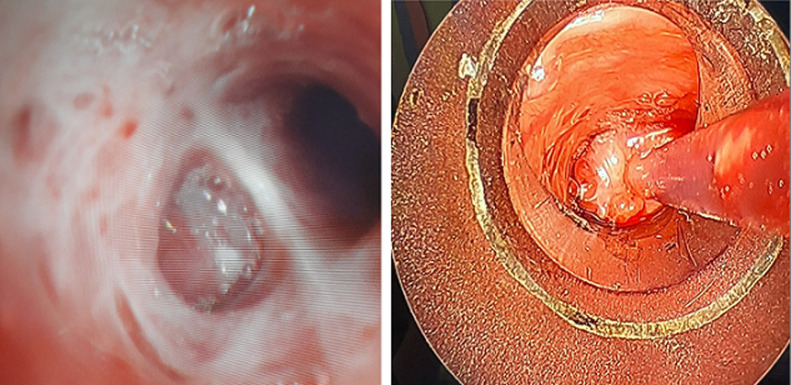
reddish, smooth tumor completely obstructing the left main bronchus in flexible bronchoscopy to the left and tumor seen on rigid bronchoscopy to the right

**Endoscopic assessment:** flexible bronchoscopy revealed a highly vascularized, reddish, smooth-surfaced tumor completely obstructing the terminal part of the left main bronchus, initially suspected to be a carcinoid tumor. Bronchial biopsies were not performed due to hemorrhagic risk. Bronchial aspirations were carried out to search for MBT using Xpert/Rif and culture techniques, which were negative. An exploratory rigid bronchoscopy was performed before surgery, showing the same findings as the flexible bronchoscopy (highly vascularized, reddish, smooth-surfaced tumor completely obstructing the terminal part of the left main bronchus, bleeding upon contact (Figure 3).

### Therapeutic intervention

**Surgical intervention:** diagnostic and therapeutic was indicated for the patient, who underwent a left pneumonectomy without lymph node dissection as there were no mediastinal lymphadenopathies. Macroscopic examination of the surgical specimen revealed a whitish, firm neoplasm measuring 27x25x25cm, with distances from the neoplasm to the bronchial extremity and pulmonary hilum of 4cm, and from the neoplasm to the pleural extremity of 3cm. The remaining lung parenchyma was fibrous with bronchiectasis and distal emphysematous bullae. Microscopic examination revealed a tumor proliferation arranged in nests, cords, and trabeculae, with large to medium-sized cells, large nuclei, mottled chromatin, visible nucleoli, and a mitotic index of 7 mitoses/2mm^2^. Immunohistochemistry showed that the tumor cells expressed chromogranin A but not CD56. The proliferation index assessed by Ki-67 was estimated at 1%. These histopathological results were consistent with an atypical carcinoid tumor. An extension work-up revealed no clinical signs of bone or abdominal involvement. The final diagnosis was an atypical carcinoid tumor with carcinoid syndrome. The case was discussed in a multidisciplinary oncothoracic meeting, and the decision was to perform a thoracoabdominal-pelvic CT scan six months post-surgery and annually thereafter, given the complete tumor resection and absence of lymphadenopathy.

**Figure 4:**
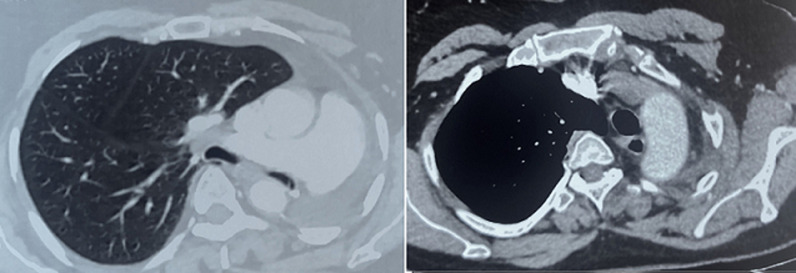
monitoring by thoraco-abdominal-pelvic CT scan after 1 year of pneumectomy

**Follow-up and outcome:** at the medium-term follow-up (six months postoperatively), our patient reported a reduction in chest pain, normalization of bowel movements, and resolution of rhythm disorders (Figure 4). During the long-term follow-up (four years post-surgery), the patient underwent a thoracic-abdominal-pelvic CT scan, which was normal, and attended regular cardiology consultations for hypertension and right bundle and consent as follows:

**Patient perspective:** the patient appreciated the surgical treatment, noting a significant improvement in her clinical symptoms and overall health

**Informed consent:** the patient gave her consent to use these clinical data for scientific purposes branch block.

## Discussion

Bronchial carcinoid tumors are neuroendocrine tumors representing 1-2% of all primary bronchopulmonary tumors. These tumors are divided into typical carcinoids (80-90%) and atypical carcinoids (10-20%) [3]. A male predominance is noted in atypical carcinoids, with a higher incidence in the fifth decade of life, as seen in our patient, and smoking is often implicated in their development [2]. The incidence of spontaneous pneumothorax in pulmonary neoplasms is generally rare, but there is no data on the incidence of pneumothorax in atypical carcinoid tumors [3]. The mechanism of pneumothorax occurrence in carcinoid tumors is similar to that in pulmonary tumors in general, distinguished by peripheral parenchymal destruction [2]. The tumor lesion protrudes into the bronchial lumen, creating a one-way valve effect, trapping inspired air in the pulmonary lobe, leading to hyperinflation of the downstream lung parenchyma, and distension of distal alveoli until their rupture into the pleural cavity [4,5]. Our patient had residual effects of tuberculosis on the same side as the pneumothorax and the carcinoid tumor, so the pneumothorax mechanism could be explained either by the carcinoid tumor mechanism or by the destruction of pulmonary parenchyma following the rupture of residual tuberculous lesions. Discovery circumstances and clinical manifestations can either present as insidious non-specific respiratory symptoms, such as dyspnea, persistent cough, and chest pain, indicating neoplastic development of the carcinoid tumor complicated by acute pain with or without desaturation, or immediately as a noisy pneumothorax with subsequent discovery of the tumor [5].

Sometimes, the carcinoid tumor can be secretory, causing carcinoid syndrome, which was seen in our patient, presenting with flushing, dyspnea, chronic diarrhea, or cardiac disorder. Thoracic CT is essential for visualizing the tumor, specifying its location, assessing the downstream pulmonary parenchyma, and identifying lymphadenopathy. The role of bronchoscopic examination is crucial as it specifies the etiology of pneumothorax and identifies a neoplastic origin by revealing an endobronchial lesion in the form of smooth and hypervascularized growth [6]. In localized atypical carcinoids, like in our patient, surgery is the gold standard, involving wide resection such as lobectomy or pneumonectomy with lymph node dissection. The surgical specimen confirms the histological type and the resection's efficacy, detailing the resection margins' histopathological aspects. Adjuvant radiotherapy or chemotherapy is recommended for locally advanced atypical carcinoids but lacks efficacy proof [5]. The follow-up recommendations suggest a check-up visit between the third and sixth months postoperatively, followed by visits every 6 to 12 months for seven years [7], monitored by Octreotide Positron Emission Tomography (PET), which was not performed in our patient due to financial constraints [8]. This clinical case is significant not only due to its rare and unexpected presentation but also because it underscores the importance of prompt and thorough diagnosis for conditions with atypical presentations. Therefore, each case of atypical carcinoid tumor is crucial for deepening our understanding of its clinical features, treatment options, and prognosis, and for contributing to the broader medical knowledge base.

## Conclusion

Current evidence on respiratory complications as indicators of carcinoid tumors is limited. Thoracic CT and bronchoscopy remain key diagnostic exams. Surgery is the gold standard for tumor resection and pneumothorax treatment. The efficacy of chemotherapy and radiotherapy has not been established.
